# IL-10-Producing Regulatory B Cells Are Decreased in Patients with Common Variable Immunodeficiency

**DOI:** 10.1371/journal.pone.0151761

**Published:** 2016-03-18

**Authors:** Nathalia Silveira Barsotti, Rafael Ribeiro Almeida, Priscilla Ramos Costa, Myrthes Toledo Barros, Jorge Kalil, Cristina Maria Kokron

**Affiliations:** 1 Laboratory of Clinical Immunology and Allergy—LIM60, Division of Clinical Immunology and Allergy, Department of Medicine, University of São Paulo School of Medicine, São Paulo, Brazil; 2 Institute for Investigation in Immunology-INCT, São Paulo, Brazil; 3 Heart Institute, (InCor), University of São Paulo School of Medicine, São Paulo, Brazil; 4 Primary Immunodeficiency Outpatient Clinic of Clinical Immunology and Allergy Division of HC-FMUSP, São Paulo, Brazil; COCHIN INSTITUTE, Institut National de la Santé et de la Recherche Médicale, FRANCE

## Abstract

Common variable immunodeficiency (CVID) is the most prevalent symptomatic primary immunodeficiency in adults. CVID patients often present changes in the frequency and function of B lymphocytes, reduced number of Treg cells, chronic immune activation, recurrent infections, high incidence of autoimmunity and increased risk for malignancies. We hypothesized that the frequency of B10 cells would be diminished in CVID patients because these cells play an important role in the development of Treg cells and in the control of T cell activation and autoimmunity. Therefore, we evaluated the frequency of B10 cells in CVID patients and correlated it with different clinical and immunological characteristics of this disease. Forty-two CVID patients and 17 healthy controls were recruited for this study. Cryopreserved PBMCs were used for analysis of T cell activation, frequency of Treg cells and characterization of B10 cells by flow cytometry. IL-10 production by sorted B cells culture and plasma sCD14 were determined by ELISA. We found that CVID patients presented decreased frequency of IL-10-producing CD24^hi^CD38^hi^ B cells in different cell culture conditions and decreased frequency of IL-10-producing CD24^hi^CD27^+^ B cells stimulated with CpG+PIB. Moreover, we found that CVID patients presented lower secretion of IL-10 by sorting-purified B cells when compared to healthy controls. The frequency of B10 cells had no correlation with autoimmunity, immune activation and Treg cells in CVID patients. This work suggests that CVID patients have a compromised regulatory B cell compartment which is not correlated with clinical and immunological characteristics presented by these individuals.

## Introduction

Common variable immunodeficiency (CVID) is the most prevalent symptomatic primary immunodeficiency in adults, characterized by hypogammaglobulinemia and defective antibody responses. The most common clinical manifestation is recurrent bacterial infections, especially in the respiratory tract [1–3]. Malignancy, chronic gastroenteropathies and autoimmunity are also often present. Autoimmunity alone may affect 20% to 50% of patients. Idiopathic thrombocytopenic purpura, autoimmune hemolytic anemia, celiac disease, atrophic gastritis, ulcerative colitis and vitiligo are the most prevalent autoimmune diseases in these individuals [2, 4–6].

Numerous cellular dysfunctions are present in CVID comprising both T and B cells, which suggest combined immune defects. Decreased frequency of naïve T cells and T_reg_ cells, increased chronic activated T cells [7–9] and altered cytokine production [10, 11] are some of the defects related to CVID. Recent reports have shown that chronic T cell activation is related to microbial translocation and increased levels of plasma sCD14 [11, 12]. CVID is also characterized by severe defects in B cell population. Besides the hallmark—hypogammaglobulinemia, the most frequent are poor antibody response to vaccines, reduction in class-switched memory B cells (CD19^+^ CD27^+^), expansion of naïve B cells as well as CD21^low^ B cells [13, 14]. However, most of CVID patients have normal or slightly reduced frequency of CD19^+^ B cells [7, 13].

B lymphocytes are predominantly associated with humoral immune responses, but other functions have been described for these cells, such as antigen presentation, inflammatory cytokine production, and, more recently, regulatory functions, performed by B_reg_ cells, which negatively modulate cell immune responses [15–17]. The absence or dysregulated function of these cells contributes to the worsening of inflammatory and autoimmune diseases [18, 19].

IL-10-producing B_reg_ cells were recently described in humans, being called B10 cells and characterized as the primary source of this cytokine. Their progenitor has been described as B10pro cells, which secrete IL-10 *in vitro* when stimulated by LPS, CpG or other TLR agonists [17, 19]. The phenotypic markers for B10 cells are not well described; yet, IL-10 production following appropriate stimulation is the best way to identify these cells [20, 21]. Some studies indicate that B10 cells are not restricted to one subpopulation and suggest human B10 cells as IL-10-producing CD24^hi^CD38^hi^ and CD24^hi^CD27^+^ B cells [17, 22–24].

The regulatory functions of B10 cells are mainly associated with their cytokine production. Through IL-10 and TGF-β production B_reg_ cells can restore T_H_1/T_H_2 balance, induce the expansion of T_reg_ cells and inhibit T_H_17 cells [25–27]. Induction of apoptotic cells and activation of macrophages, dendritic cells and iNKT cells are also related directly or indirectly to B10 cells [25]. The role of B10 cells in inflammatory diseases, cancer and autoimmunity has been well characterized in animal models, but few studies in humans have been performed [28].

The fact that CVID patients often present alterations in B lymphocytes, reduced number of T_reg_ cells and chronic immune activation, as well as high incidence of autoimmunity, suggests that the frequency of B10 cells may be diminished in these individuals, since such cells play an important role in the development of T_reg_ cells and in the control of T cell activation and autoimmunity. Recently, Vlkova *et al*. [29] showed that CVID patients present reduced frequency of CD24^hi^CD38^hi^ IL-10-producing B cells. However, Kofod-Olsen *et al*. [30] demonstrated that pro-B10 cells are increased in CVID patients. Therefore, this study proposes an analysis of the frequency of two B10 cell subtypes (CD24^hi^CD38^hi^ and CD24^hi^CD27^+^) in CVID patients and its relation to immunological changes previously described in these individuals in order to contribute to a better understanding of common variable immunodeficiency.

## Materials and Methods

### Study population

In this study 42 CVID patients (median age 32, range 23–58 years) from Primary Immunodeficiency Outpatient Clinic of Clinical Immunology and Allergy Division of HC-FMUSP, fulfilling the PAGID / ESID criteria (1999) for CVID diagnosis, and 17 healthy individuals (median age 28, range 23–46) were recruited. The study was approved by Hospital das Clínicas, University of São Paulo Medical School Ethics Committee (CAPPesq) and all patients and healthy volunteers signed the informed consent before inclusion into the study.

### Human Cell Isolation

Peripheral blood mononuclear cells (PBMC) were isolated from fresh heparinized blood by Ficoll-Paque^TM^ Plus (Invitrogen) density gradient centrifugation. Isolated PBMC were resuspended in FCS (GIBCO) with 10% of DMSO (GIBCO) at a concentration of 5 x 10^6^ cells/ml and frozen until subsequent use.

### Flow cytometry analysis of regulatory B cells

PBMC (1x10^6^ cells/ml) were cultured in complete medium (RPMI 1640, Invitrogen) supplemented with 10% FCS (Gibco), 1mM sodium pyruvate (Gibco), 200 mg/ml penicillin (Gibco), 200 U/ml streptomycin (Gibco), 4mM L-glutamine (Gibco) and 0.1% 2- mercaptoethanol (Gibco) in the presence of CpG (ODN 2006, 10 μg/ml; Invivogen) or LPS (10 μg/ml, *Escherichia coli*; Sigma-Aldrich), PMA (50 ng/ml; Sigma-Aldrich), ionomycin (1 μg/ml; Sigma-Aldrich) and brefeldin A (BFA; 1μl/ml; Golgi Plug^®^; BD Biosciences) in 96-well U-bottom plates for 5h, at 37°C (PIB: PMA + ionomycin + brefeldin A). For analysis of B10 cells by flow cytometry, cultured cells were stained for viable cells using LIVE/DEAD fixable red dead cell stain kit (Invitrogen) and with the following anti-human mAb (all from BD Biosciences): CD3 (UCHTI, PE-CF594), CD14 (TüK4, PE-CF594), CD19 (HIB19, APC), CD24 (ML5, PerCP-Cy5.5), CD27 (M-T271, PE-Cy7), CD38 (HIT-2, FITC). For intracellular IL-10 analysis, surface-stained cells were fixed/permeabilized using Fix/Perm kit (BD Biosciences) and stained with anti-human IL-10 (JES3-19F1, PE, BD Biosciences). Flow cytometry was performed using FACS Canto II (BD Biosciences) and the data was analyzed using FlowJo^®^ software (Tree Star). The gate strategy used to determine IL-10 expression was designed based on cells cultured in the presence of BFA only.

### Production of IL-10 by B cells

IL-10 production by B cells was determined in supernatant of sorting-purified B cells cultured with CpG and CD40L for 48 hours, using the *Quantikine*^*®*^ *ELISA—Human IL-10 Immunoassay* (R&D Systems). PBMC’s were stained for viable cells using LIVE/DEAD fixable red dead cell stain kit (Invitrogen) and with the following anti-human mAb (all from BD Biosciences): CD3 (UCHTI, PE-CF594), CD14 (TüK4, PE-CF594) and CD19 (HIB19, APC). CD3 and CD14 staining were used as an exclusion gate. Viable CD19^+^ B cells were sorted using FACS Aria IIu (BD Bioscience), plated in 96-well U-bottom plates at 2.5 X 10^5^ cells/well and cultured in complete medium (RPMI 1640, Invitrogen) supplemented with 10% FCS (Gibco), 1mM sodium pyruvate (Gibco), 200 mg/ml penicillin (Gibco), 200 U/ml streptomycin (Gibco), 4mM L-glutamine (Gibco) and 0.1% 2- mercaptoethanol (Gibco) in the presence of CpG (ODN 2006, 10 μg/ml; Invivogen) and CD40L (1μg/ml; Sigma-Aldrich) for 48 hours at 37°C. The supernatant was collected and cryopreserved. Supernatant samples were diluted 10-fold with diluent solution prior to analysis. Optical density was determined using a Microplate Reader (Bio-Tek) and IL-10 concentrations were calculated using the Gen5 (Bio-Tek).

### Flow cytometry analysis of activated T cells

PBMC (1x10^6^ cells/ml) were stained for viable cells using LIVE/DEAD fixable acqua dead cell stain kit (Invitrogen) and with the following anti-human mAb (all from BD Biosciences): CD3 (UCHTI, PE-CF594), CD4 (RPA-T4, APC-Cy7), CD8 (SK1, PerCP-Cy5.5), CD69 (FN50, PE), CD38 (HIT-2, FITC) and HLA-DR (G46-6, Alexa Fluor 700). Flow cytometry was performed using LSR Fortessa (BD Biosciences) and the data was analyzed using FlowJo^®^ software (Tree Star).

### Flow cytometry analysis of T_reg_ cells

PBMC (1x10^6^ cells/ml) were stained for viable cells using LIVE/DEAD fixable acqua dead cell stain kit (Invitrogen) and with the following anti-human mAb (all from BD Biosciences): CD3 (UCHTI, PE-CF594), CD4 (RPA-T4, APC-Cy7), CD8 (SK1, PerCP-Cy5.5), CD25 (M-A251, PE), CD127 (HIL-7R-M21, FITC) and CD39 (A1, PE-Cy7). For intranuclear Foxp3 analysis, surface-stained cells were fixed and permeabilized according to the manufacturer’s instructions using Human FoxP3 Buffer Set kit (BD Biosciences) and stained with anti-human Foxp3 (259D/C7, Alexa Fluor 647; BD Biosciences). Flow cytometry was performed using a LSR Fortessa (BD Biosciences) and the data was analyzed using FlowJo^®^ software (Tree Star).

### Human sCD14 assay

Plasma sCD14 levels were determined using the Human sCD14 Quantikine^®^ ELISA (R&D Systems). Plasma samples were diluted 200-fold with sample diluent solution prior to analysis. Optical density was determined using a Microplate Reader 3550 (BioRad) and plasma sCD14 concentrations were calculated using the Microplate Manager 4.0 (BioRad).

### Statistical Analysis

Statistical analysis was performed using GraphPad Prism version 6.0 (GraphPad Software). Results are expressed as mean ± SEM. Experimental groups were compared using a two-tail Mann-Whitney test. For correlation analysis Spearman’s test was used. *P* values <0.05 were considered statistically significant.

## Results

### Study population

Clinical and laboratory characteristics of CVID patients are shown in Table 1 and Table 2. CVID patients show a variety of recurrent infections, being respiratory infections the most prevalent. Seventeen (17) CVID patients also present autoimmune diseases. As expected, CVID patients showed a reduced number of total lymphocytes, reduced number of CD4^+^ T cells and increased number of CD8^+^ T cells. Also, the frequency of CD19^+^ B cells was similar in patients and healthy controls. Patients with low number of B cells (<1% of PBMC) were excluded from this study.

**Table 1:** Clinical characteristics of CVID patients and healthy controls.

Subjects	Healthy controls	CVID
**N**	17	42
**Gender**		
Male	7	21
Female	10	21
**Age (years range)**	23–46	23–58
**Clinical characteristics of CVID patients**		
**Recurrent infections**		
Pneumonia		34/42
Sinusitis		23/42
Otitis		3/42
Tonsillitis		1/42
URTI		2/42
Diarrhea		15/42
Meningitis		1/42
**No autoimmunity**		25/42
**Autoimmunity**		17/42
**Cytopenias**		
Hemolytic anemia		1/17
ITP		4/17
**Gastrointestinal Diseases**		
Atrophic Gastritis		6/17
Ulcerative Colitis		1/17
Celiac Disease		3/17
**Endocrine Diseases**		
Diabetes Mellitus		1/17
Hypothyroidism		3/17
**Skin Diseases**		
Psoriasis		1/17
Vitiligo		2/17
Vasculitis		2/17

**Table 2:** Laboratory characteristics of CVID patients and healthy controls.

	Healthy Controls	CVID	
	(N = 17)	(N = 42)	
	Median		*p
**Lymphocytes (mil/mm**^**3**^**)**	2.25 (1.36–3.34)	1.66 (0.29–3.24)	0.0128*
**CD3**^**+**^ **T (mil/mm**^**3**^**)**	1.70 (0.80–2.60)	1.30 (0.26–2.60)	0.0906
**CD4**^**+**^ **T (mil/mm**^**3**^**)**	0.90 (0.44–1.55)	0.55 (0.09–1.35)	0.0001*
**CD8**^**+**^ **T (mil/mm**^**3**^**)**	0.43 (0.18–0.82)	0.59 (0.14–1.51)	0.2741
**Ratio CD4**^**+**^ **T/ CD8**^**+**^ **T**	1.80 (1.00–5.00)	0.59 (0.14–1.51)	0.0005*
**CD19**^**+**^ **B (mil/mm**^**3**^**)**	0.17 (0.09–0.41)	0.09 (0.01–0.61)	0.0657

*p = Mann-Whitney test.

### CVID patients have decreased frequencies of IL-10-producing B cells

**Fig 1:**
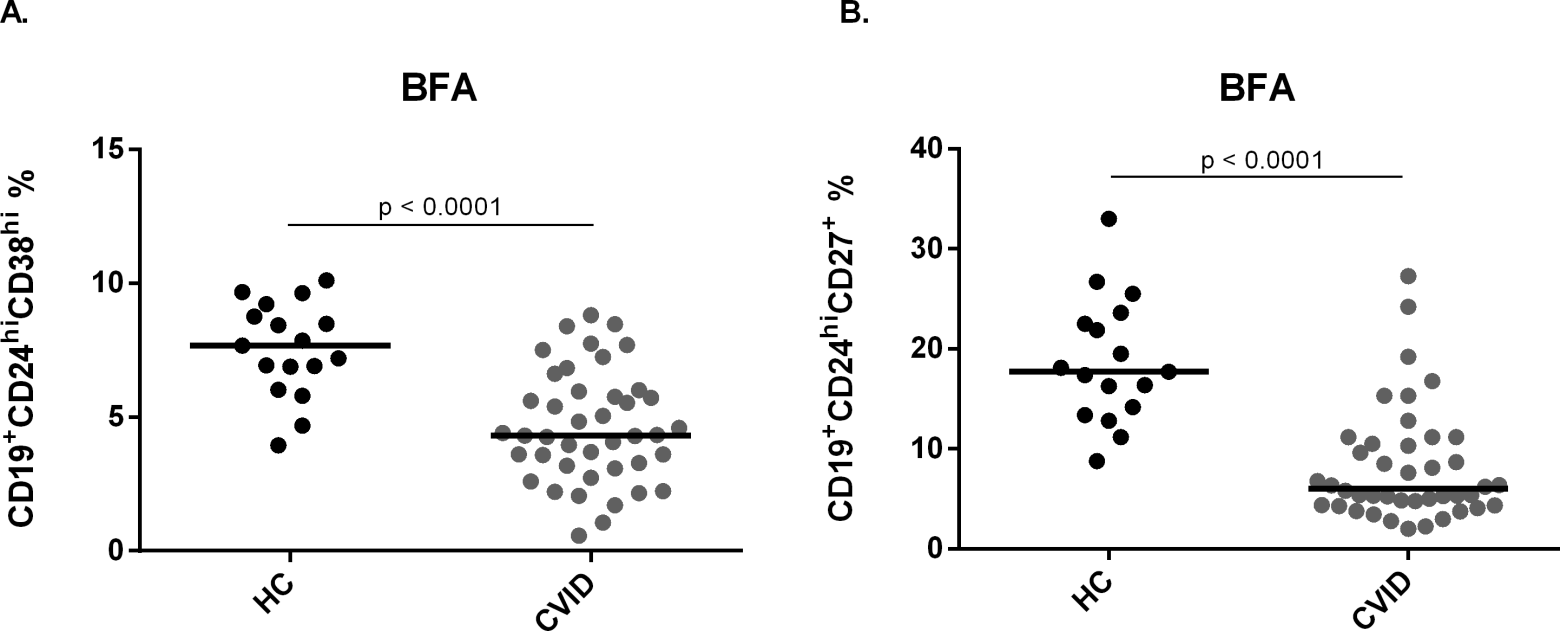
Frequency of CD24^hi^CD38^hi^ and CD24^hi^CD27^+^ cells in healthy controls and CVID patients. B cell subsets were determined by the expression of surface markers CD24, CD38 and CD27, and analyzed by flow cytometry. Scatter plots show the percentages of B cell subsets (A) CD19^+^CD24^hi^CD38^hi^ and (B) CD19^+^CD24^hi^CD27^+^ in peripheral blood of 17 healthy controls (HC) and 42 CVID patients. Bars represent mean value. *P* values were calculated by Mann-Whitney test.

Human B10 cells have been characterized as IL-10-producing CD24^hi^CD38^hi^ and CD24^hi^CD27^+^ subsets. To determine the frequency of these subpopulations in CVID patients we stained PBMC with specific antibodies for flow cytometry analysis (**S1 Fig**). We first evaluated the frequency of CD24^hi^CD38^hi^ and CD24^hi^CD27^+^ cells disregarding IL-10 production and observed that both subsets are significantly decreased in CVID patients when compared with healthy controls (p< 0.0001) (**Fig 1**).

**Fig 2:**
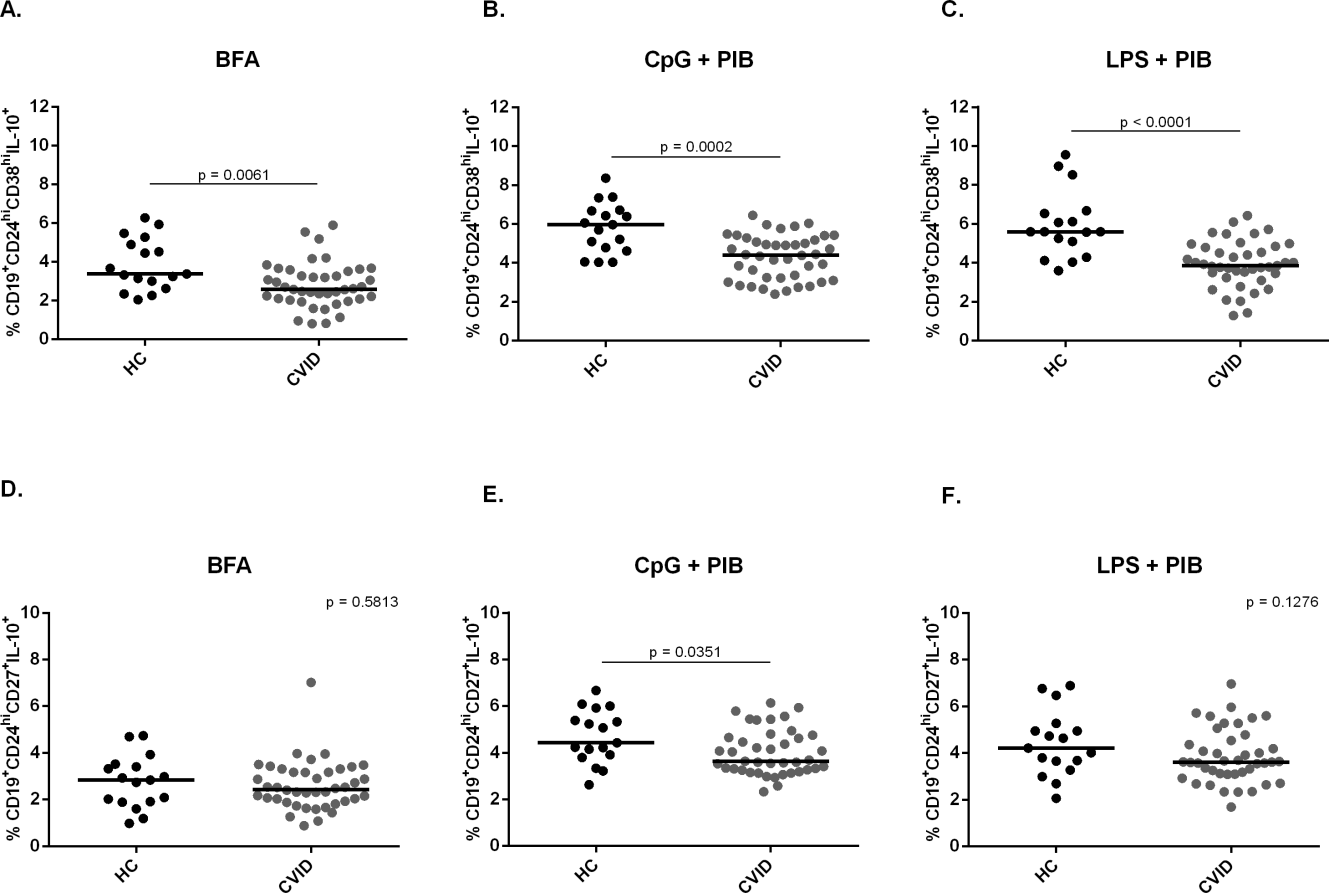
Frequency of CD24^hi^CD38^hi^ and CD24^hi^CD27^+^ B10 cells in healthy controls and CVID patients. The expression of intracellular IL-10 and surface markers CD24, CD38 and CD27 were determined by flow cytometry after *in vitro* stimulation of PBMC for 5h with only BFA; CpG + PIB or LPS + PIB. Scatter plots show the percentages of B10 cell subsets (A,B,C) CD19^+^CD24^hi^CD38^hi^IL-10^+^ and (D,E,F) CD19^+^CD24^hi^CD27^+^IL-10^+^ in peripheral blood of 17 healthy controls (HC) and 42 CVID patients. Bars represent the mean value. *P* values were calculated by Mann-Whitney test.

We then evaluated the frequency of IL-10-producing CD24^hi^CD38^hi^ and CD24^hi^CD27^+^ cells in different cell culture conditions; only BFA, CpG+PIB or LPS+PIB for 5h incubation. We found that in all conditions the frequency of CD24^hi^CD38^hi^IL-10^+^ cells was significantly reduced in CVID patients when compared to healthy controls (p = 0.0061; p = 0.0002; p< 0.0001, respectively) (**Fig 2A**). Moreover, we found a significantly decreased frequency of CD24^hi^CD27^+^ B10 subset in the condition with CpG+PIB (p = 0.0351) (**Fig 2B**). Therefore, these results suggest that CVID patients present a decreased frequency of B10 cells when compared with healthy controls.

### CVID patients present lower IL-10 production by B cells than healthy controls

**Fig 3:**
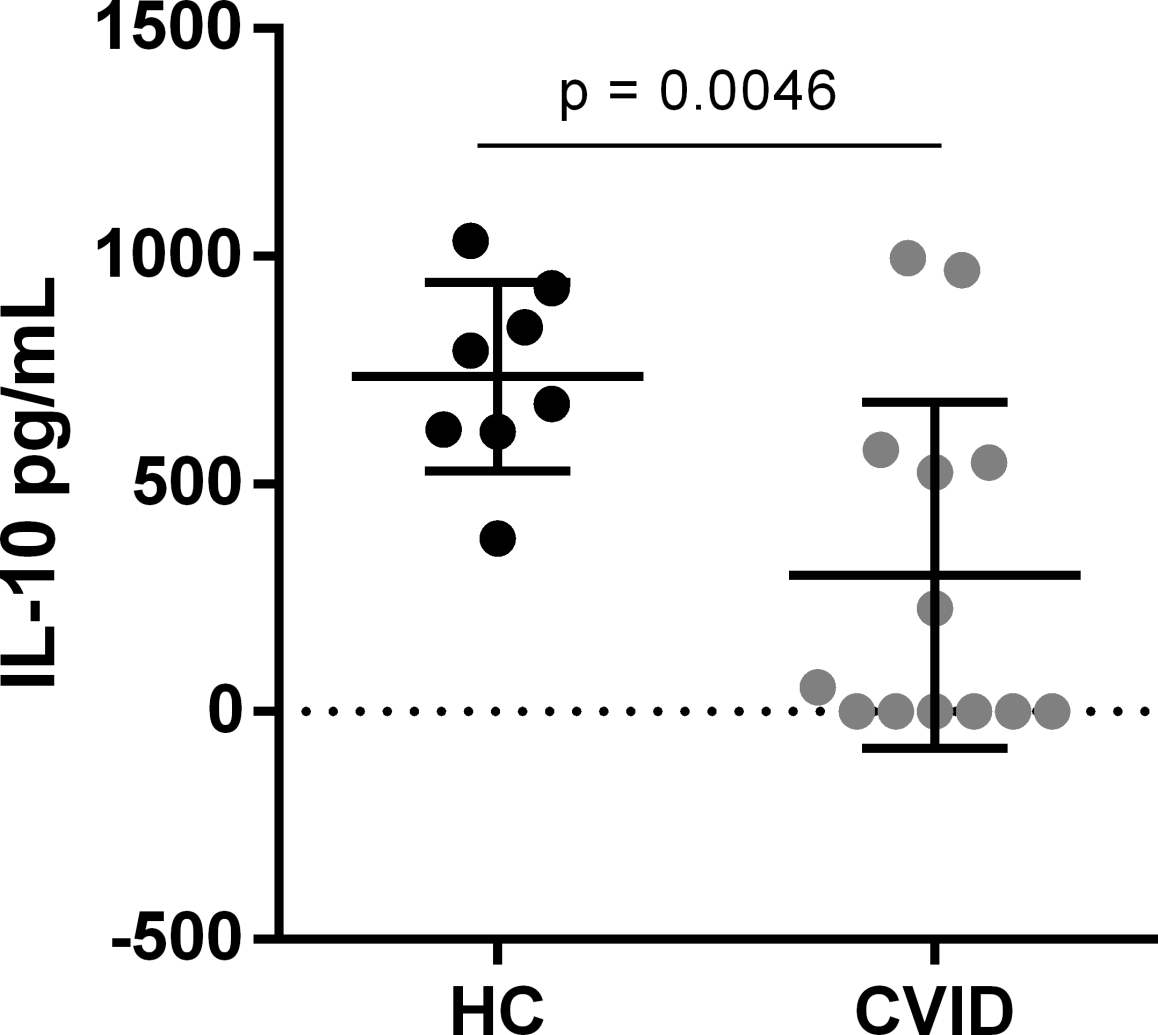
IL-10 production by B cells from CVID patients and healthy controls. IL-10 production from sorting-purified B cells of 9 healthy controls (HC) and 13 CVID patients was determined in the supernatant of cultures with CpG and CD40L for 48 hours by ELISA. Bar graphs represent mean IL-10 production (± SEM). *P* values were calculated by Mann-Whitney test.

In order to further characterize B10 cells from CVID patients, we evaluated IL-10 production by sorting-purified B cells. We found a significant decrease in IL-10 production by B cells from CVID patients when compared to healthy controls (p = 0.0046) (**Fig 3**). Interestingly, almost half of CVID patients presented no production of IL-10 by B cells. Therefore, these data suggest that CVID patients may have a compromised production of IL-10 by B cells.

### CVID patients with autoimmune cytopenias present decreased frequency of B10 cells

**Fig 4:**
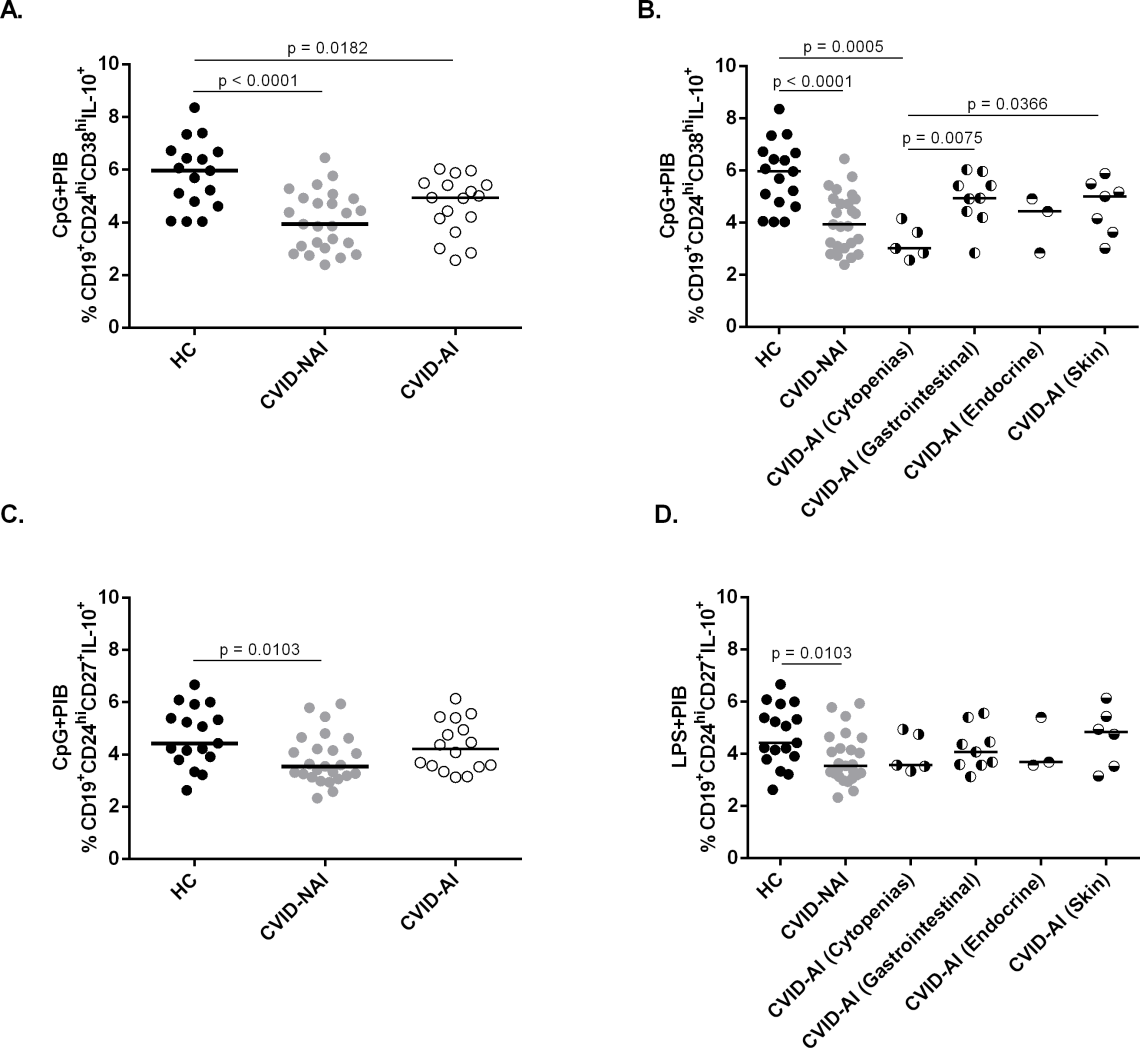
Frequency of IL-10-producing CD24^hi^CD38^hi^ and CD24^hi^CD27^+^ B cells in CVID-NAI and CVID-AI patients. The expression of intracellular IL-10 and surface markers CD24, CD38 and CD27 were determined by flow cytometry after *in vitro* stimulation of PBMC for 5h with CpG + PIB. Scatter plots show the percentages of (A,B) CD19^+^CD24^hi^CD38^hi^IL-10^+^ and (C,D) CD19^+^CD24^hi^CD27^+^IL-10^+^ B10 cells in peripheral blood of 17 Healthy Controls (HC), 25 CVID-NAI and 17 CVID-AI patients. Bars represent the mean value. *P* values were calculated by Mann-Whitney test.

Due to the fact that CVID patients present high prevalence of autoimmune diseases, we analyzed patients separately as CVID with autoimmunity (CVID-AI) and without autoimmunity (CVID-NAI). We observed that CVID-AI patients present no significant difference in the frequency of CD24^hi^CD38^hi^ and CD24^hi^CD27^+^ B10 cells when compared with CVID-NAI (**Fig 4A and 4C**). However, when we analyzed the autoimmunity group divided as cytopenias and gastrointestinal diseases, we found a decreased frequency of CD24^hi^CD38^hi^ B10 cells in CVID patients with autoimmune cytopenias when compared to CVID patients with gastrointestinal autoimmune diseases (p = 0.0075) and to healthy controls (p = 0.0005) (**Fig 4B**). The same was not found regarding CD24^hi^CD27^+^ B10 cells (**Fig 4D**). Taken together, these data suggest that reduction of B10 cells may have a differential impact on autoimmune manifestation in CVID patients.

### Chronic activated T cells and sCD14 plasma levels have no correlation with B10 cells in CVID patients

**Fig 5:**
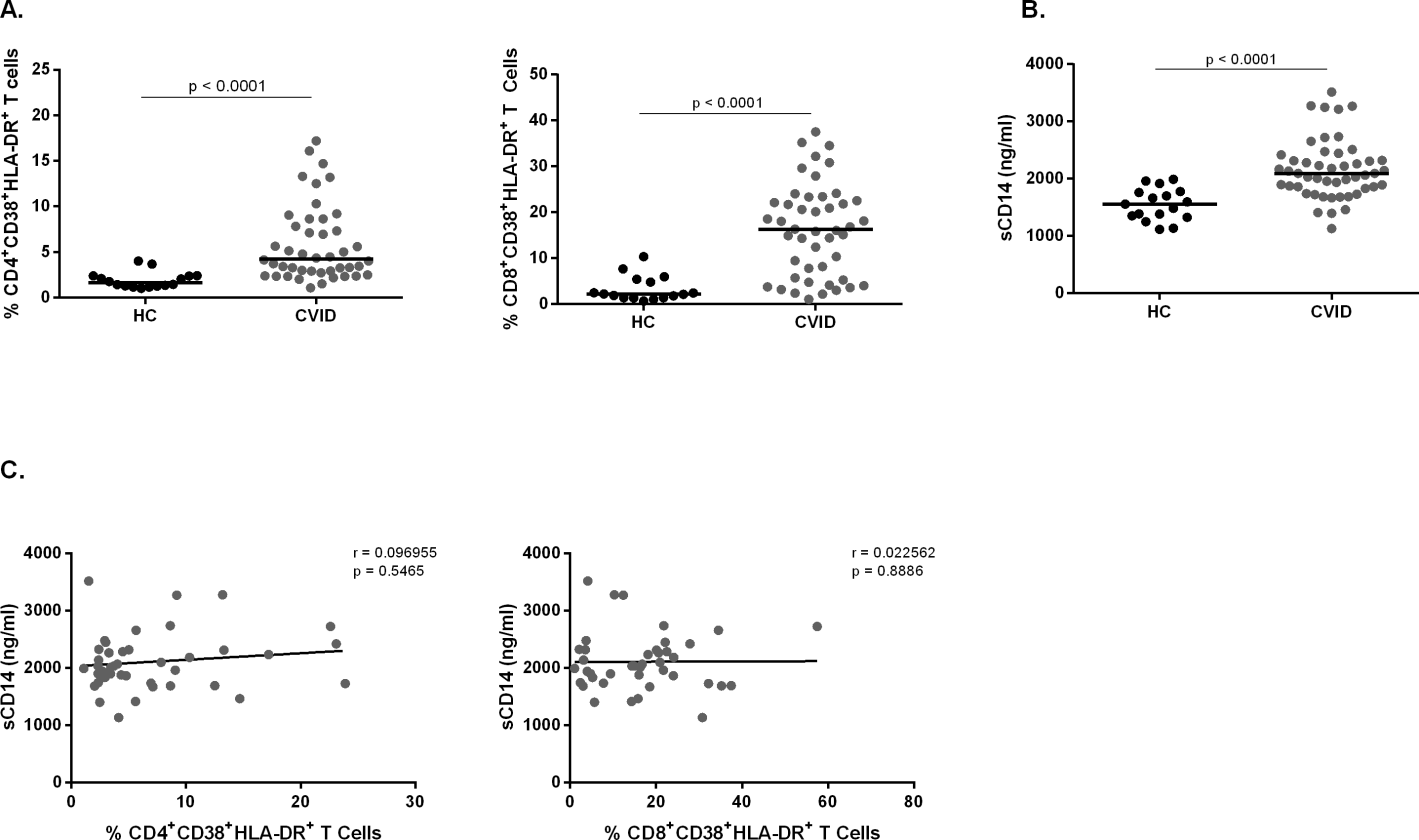
Chronic activation profile in CVID patients. Chronic activated T cells were determined in PBMC from 17 healthy controls and 42 CVID patients by surface markers CD4, CD8, CD38 and HLA-DR expression without stimulation and analyzed by flow cytometry. sCD14 levels were determined by ELISA analysis of plasma from 17 healthy controls and 42 CVID patients. Scatter plots show the percentages of **(A)** CD4^+^CD38^+^HLA-DR^+^ and CD8^+^CD38^+^HLA-DR^+^ chronic activated T cells and **(B)** plasma levels of sCD14. **(C)** Correlation of sCD14 with the frequencies of chronic activated CD4^+^ and CD8^+^ T cells in CVID patients. Bars represent the mean value. *P* values were calculated by Mann-Whitney test. Lines represent linear regression analysis. *R* and *P* values were calculated by Spearman’s rank order correlation test.

It has been shown that CVID patients present higher frequency of activated T cells and increased plasma sCD14 levels [12]. Therefore, we intended to investigate whether these parameters would correlate with B10 cell frequency. We evaluated the frequency of CD4^+^CD38^+^HLA-DR^+^ and CD8^+^CD38^+^HLA-DR^+^ T cells by flow cytometry, and plasma sCD14 levels by ELISA and correlated them with B10 cells. As expected, we found higher frequencies of chronic activated CD4^+^ and CD8^+^ T cells and increased plasma sCD14 levels in CVID patients when compared with healthy controls (**Fig 5A and 5B**), although no correlation between sCD14 and chronic activated CD4^+^ and CD8^+^ T cells was found (**Fig 5C**).

**Fig 6:**
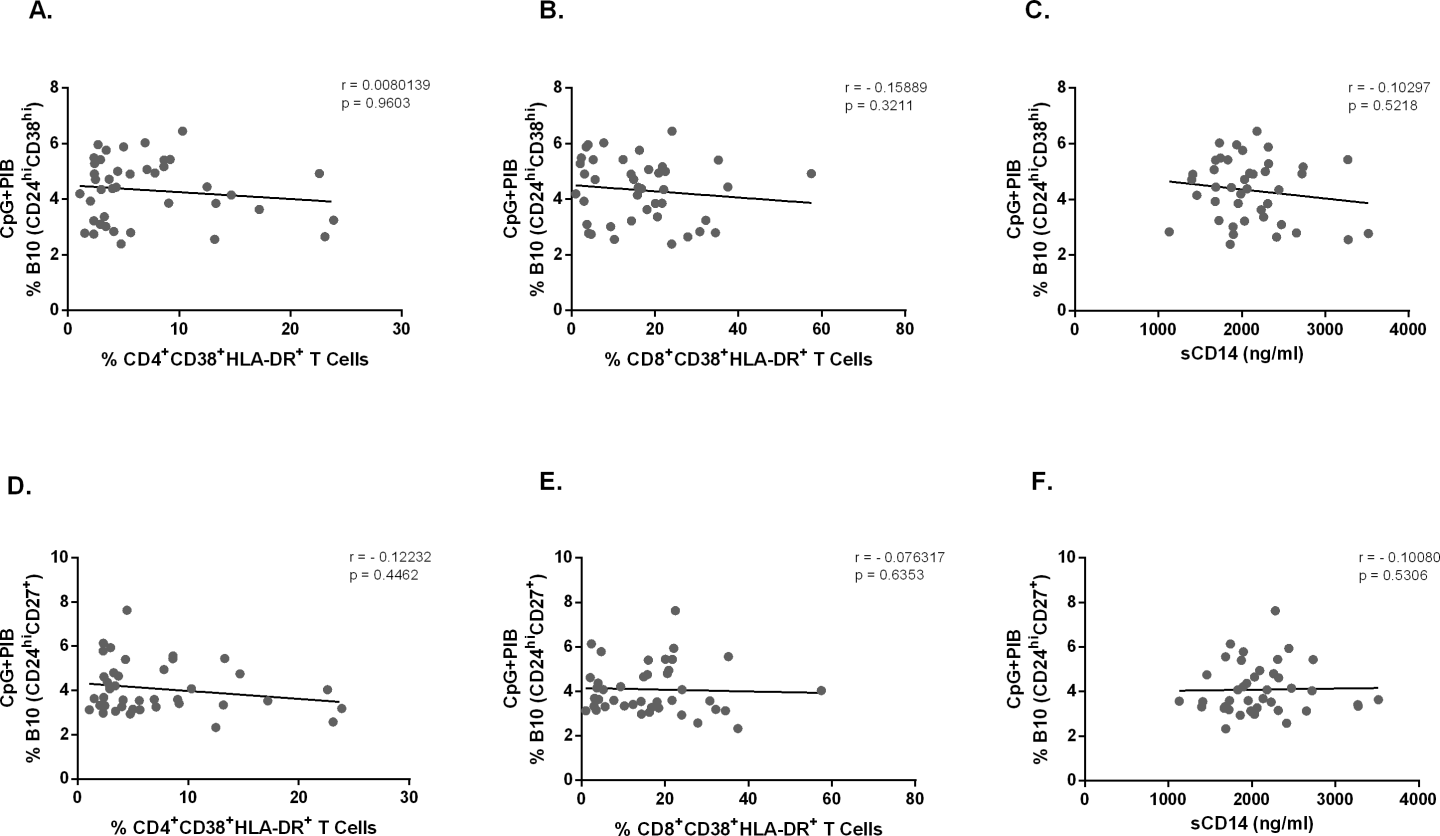
Correlation analysis of B10 cells and chronic activated T cells. The expression of intracellular IL-10 and surface markers CD24, CD38 and CD27 were determined by flow cytometry after *in vitro* stimulation of PBMC for 5h with CpG + PIB. Chronic activated T cells were determined in PBMC by the expression of surface markers CD4, CD8, CD38 and HLA-DR without stimulation and analyzed by flow cytometry. sCD14 levels in plasma were determined by ELISA. Correlation of CD19^+^CD24^hi^CD38^hi^ IL-10^+^ cells with chronic activated (A) CD4^+^ and (B) CD8^+^ T cells in 43 CVID patients. Correlation of CD19^+^CD24^hi^CD27^+^ IL-10^+^ cells with chronic activated (D) CD4^+^ and (E) CD8^+^ T cells in 43 CVID patients. Lines represent linear regression analysis. Correlation of CD19^+^CD24^hi^CD38^hi^ IL-10^+^ (C) and CD19^+^CD24^hi^CD27^+^IL-10^+^ (F) B cells with plasma levels of sCD14 in 41 CVID patients. Lines represent linear regression analysis. *R* and *P* values were calculated by Spearman’s rank order correlation test.

We then evaluated whether these parameters would correlate with the frequency of CD24^hi^CD38^hi^ and CD24^hi^CD27^+^ B10 cells in CVID patients and found no correlation (**Fig 6**). Overall, these data suggest that both T cell activation and plasma sCD14 levels have no correlation with the frequency of B10 cells in CVID patients.

### The frequency of B10 cells has no correlation with the frequency of T_reg_ cells in CVID patients

**Fig 7:**
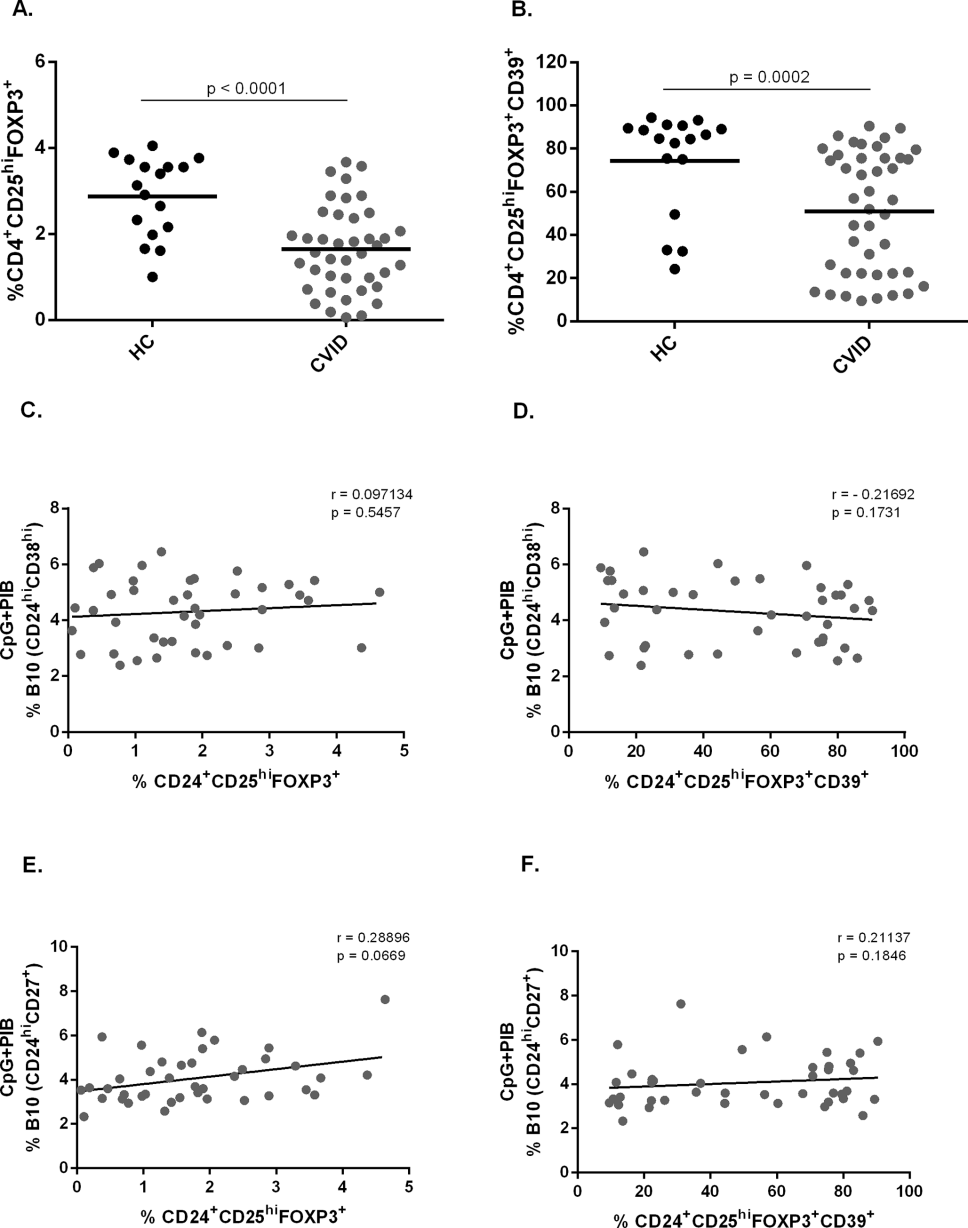
Frequency of T_reg_ cells and CD39^+^T_reg_ cells in CVID patients and correlation analysis of B10 cells with T_reg_ cells and CD39^+^T_reg_ cells. Foxp3 and surface markers CD4, CD25 and CD39 expression were determined by flow cytometry in peripheral blood from 17 healthy controls (HC) and 42 CVID patients. The expression of intracellular IL-10 and surface markers CD24, CD38 and CD27 was determined in B cells by flow cytometry after in vitro stimulation of PBMC for 5h with CpG (10μg/ml) + PIB. Scatter plots show the percentages of **(A)** T_reg_ cells and **(B)** CD39^+^ cells within T_regs_ in peripheral blood of 17 healthy controls (HC) and 42 CVID patients. Bars represent the mean value. *P* values were calculated by Mann-Whitney test. Correlation of **(C)** CD24^hi^CD38^hi^ and **(E)** CD24^hi^CD27^+^B10 cells with the frequency of T_reg_ cells. Correlation of **(D)** CD24^hi^ CD38^hi^ and **(F)** CD24^hi^ CD27^+^ B10 cells with CD39^+^ T_reg_ cells in 41 CVID patients. Lines represent linear regression analysis. *R* and *P* values were calculated by Spearman’s rank order correlation test.

CVID patients are known to have lower frequency of T_reg_ cells [9, 31, 32]. We therefore evaluated the frequency of these cells by flow cytometry and asked whether it would correlate with the frequency of CD24^hi^CD38^hi^ and CD24^hi^CD27^+^ B10 cells. As expected, we found a reduced frequency of CD4^+^CD25^hi^Foxp3^+^ T_reg_ cells in CVID patients when compared with healthy controls (**Fig 7A**). We then evaluated the frequency of CD39^+^ cells within CD4^+^CD25^hi^Foxp3^+^ T_reg_ cells and also found a reduced frequency in CVID patients (**Fig 7B**). We have found no difference in the frequency of CD4^+^CD25^hi^Foxp3^+^CD39^+^ T_reg_ cells in CVID patients with or without autoimmunity (data not shown). No correlation between CD24^hi^CD38^hi^ B10 and either CD4^+^CD25^hi^Foxp3^+^ T_reg_ cells or CD39^+^ T_reg_ cells was found in CVID patients (**Fig 7C and 7D**). The same was observed for CD24^hi^CD27^+^ B10 cells (**Fig 7E and 7F**). Taken together, these data suggest that although the frequencies of T_reg_ and B10 cells are both decreased in CVID patients, they have no correlation.

## Discussion

In this study, we show that CVID patients have decreased frequencies of IL-10-producing CD24^hi^CD38^hi^ B cells in different cell culture conditions and decreased frequency of IL-10-producing CD24^hi^CD27^+^ B cells stimulated with CpG+PIB, when compared with healthy controls. Furthermore, we found that CVID patients presented lower secretion of IL-10 by sorting-purified B cells when compared to healthy controls. We also show that the frequency of B10 cells obtained by 5h stimulation had no correlation with immune activation, autoimmunity and T_reg_ cells in CVID patients.

Here, we observed a decreased CD24^hi^CD38^hi^ as well as CD24^hi^CD27^+^ B cells in CVID patients. In fact, these individuals are known for having B cell defects as hypogammaglobulinemia and defective antibody responses, despite presenting normal or near normal frequency of CD19^+^ B cells [13]. CVID patients present an increased frequency of naïve B cells as well as CD21^low^ B cells and decreased frequency of class-switched memory B cells (CD19^+^CD27^+^IgD^-^) as the most frequent abnormalities among B cell populations [6, 13, 33]. Our data suggest that CVID patients may have a compromised regulatory B cell compartment, described as the main source of IL-10 in humans, due to the lower frequency of CD24^hi^CD38^hi^ and CD24^hi^CD27^+^ B cell subpopulations [22, 23, 27, 34].

Given the fact that CD24^hi^CD38^hi^ and CD24^hi^CD27^hi^ B cells were reduced in CVID patients we assessed IL-10 production in these subsets and found that CVID patients have decreased frequencies of IL-10-producing CD24^hi^CD38^hi^ B cells in the different cell culture conditions and decreased frequency of IL-10-producing CD24^hi^CD27^+^ B cells stimulated with CpG+PIB. Several studies have demonstrated that CpG is the most efficient stimulus for inducing regulatory B cells [17, 23, 27]. However, LPS has also been shown to be effective in activating B10 cells [17]. A study has demonstrated that CVID patients have a reduced frequency of CD24^hi^CD38^hi^ B10 cells [29], which is in accordance with our results. However, that study had not addressed the CD24^hi^CD27^+^ B10 cells, which has also been described as a B10 cell subtype [17]. More recently, Kofod-Olsen *el al*. [30] found elevated frequency of CD19^+^ B10 cells in CVID patients, which was correlated with some clinical conditions presented by these patients. While we have studied two subsets of B10 cells stimulated for 5h, Kofod-Olsen *el al*. evaluated IL-10-expressing B cells from 48h stimulated cultures, which may have led to the divergent result. Furthermore, differences in the incidence and type of autoimmunity presented by patients from both studies may have also accounted for the observed discrepancy. Therefore, one may notice that the profile and function of B10 cells in CVID is still uncertain. Here, we sought to evaluate the frequency of different subtypes of B10 cells and their possible correlations with clinical and immunological parameters of CVID patients such as autoimmunity, T-cell activation and regulatory T cells, which were not previously addressed. Therefore, we believe that our study has brought new insights into the characterization of B10 cells in CVID disorder.

We found that IL-10 production by B cells from CVID patients was lower when compared with healthy controls, suggesting that CVID patients have a compromised production of this cytokine by B cells. Interestingly, almost half of CVID patients tested in our study produced no detectable amounts of IL-10. Iwata *et al*. [17] has previously demonstrated that the mean of IL-10 production by B cells from healthy individuals was around 500 pg/mL, using the same approach of our study. Nonetheless, Bouaziz *et al*. [27], using CpG-B and anti-Ig for 48h of culture, demonstrated a higher mean of IL-10 production by B cells from healthy individuals, which was approximately 2000 pg/mL. We found that cells from healthy individuals produced 736.3 pg/mL of IL-10, in accordance with results from Iwata *et al*. Regarding our results, we believe that B10 cells from CVID patients may be less effective in producing IL-10 than those from healthy controls. Vlkova *et al*. [29] have demonstrated that CVID B10 cells failed to suppress IFN-γ/ TNF-α-producing CD4^+^ T cells, which may corroborate our suggestion [22, 35].

Recent findings indicate that B10 cells have both altered numbers and function in various human autoimmune conditions as rheumatoid arthritis [23], Grave’s disease [24], idiopathic thrombocytopenic purpura (ITP) [36] and systemic lupus erythematosus [22]. Moreover, it has been described that 20–50% of CVID patients develop autoimmunity [1, 4]. We therefore investigated whether autoimmunity and B10 cells would have any correlation in CVID patients and found no difference between individuals with or without autoimmune diseases. Nonetheless, we observed a reduced frequency of CD24^hi^CD38^hi^ B10 cells in CVID patients with autoimmune cytopenias, while gastrointestinal autoimmune diseases presented the frequency of B10 cells similar to healthy controls, suggesting that B10 cells may have a differential impact on the different autoimmune manifestations in CVID patients, which could be better demonstrated with a bigger cohort. According to our findings, Li *et al*., 2012 [36] have shown a decreased frequency of CD24^hi^CD38^hi^ B10 cells in non-CVID patients with chronic ITP, which suggests that these cells play a role in cytopenias independently of CVID disorders.

As demonstrated in this study, the frequency of chronic activated T cells and sCD14 plasma levels were higher in CVID patients when compared with healthy controls, with similar magnitudes as those presented in previous reports [1, 12]. However, we have not found any correlation between B10 cells and either chronic activated T cells or sCD14 plasma levels in CVID patients, suggesting that B_reg_ dysfunction in these individuals may have other causes or consequences than chronic immune activation. Differently, a positive correlation between chronic activated T lymphocytes and B_reg_ cells has been found in HIV patients [34]. We conceive that immune particularities of both diseases may have led to distinct results. To the best of our knowledge no other study has addressed a correlation between B10 cells and the parameters evaluated here. It is noteworthy that the correlations made in this study were based on 5 hours stimulated B10 cells. Stimulation for longer periods with TLR agonists may lead to different results as it takes in account B10 cells induced from progenitors (B10pro).

In this report we have demonstrated a significant reduction in the frequency of CD4^+^CD25^hi^Foxp3^+^ T_reg_ in CVID patients, as previously described [1, 9] and we were the first to show a reduced frequency of CD39-expressing CD4^+^CD25^hi^Foxp3^+^ T_reg_ in these patients. Although CD39^+^ T_reg_ cells have been claimed to be involved in the control of inflammatory autoimmune diseases [37], we have found no difference in the frequency of these cells in CVID patients with or without autoimmunity.

Due to the fact that B_reg_ cells are important for T_reg_ induction [16, 25], we evaluated whether these parameters would correlate in CVID patients. Although we have observed reduced frequencies of B10 cells and T_reg_ cells in CVID patients, no correlation was found. We have also noted no correlation between B10 cells and CD39^+^ T_reg_ cells in CVID patients. We believe that immune dysfunctions presented by CVID patients in both T and B cell compartments may be responsible for the absence of correlation observed in this study.

## Conclusions

We present here a report on the profile of B10 cells in CVID disorder. This work suggests that CVID patients have a compromised regulatory B cell compartment, which was not correlated with clinical and immunological characteristics presented by these individuals. The frequency of B10 cells can be assessed by many approaches which we believe may have an impact on how these cells correlate with parameters evaluated in this work. However, we believe that our findings might offer valuable information to better understand immune dysfunctions related to CVID.

## Supporting Information

S1 Fig Representative flow cytometry plot showing the gating strategy used to identify B10 cells. The expression of intracellular IL-10 and surface markers CD24, CD38 and CD27 were determined by flow cytometry after in vitro stimulation of PBMC for 5h with only BFA; CpG (10μg/ml) + PIB or LPS (10μg/ml) + PIB. This cytometry plot shows **(A)** CD19^+^CD24^hi^CD38^hi^ and **(B)** CD19^+^CD24^hi^CD27^+^ B10 cells after CpG + PIB stimulation.
(PDF)
