# Supplementary material for: IL-10-Producing Regulatory B Cells Are Decreased in Patients with Common Variable Immunodeficiency
Source: PLoS One. 2016 Mar 18;11(3):e0151761. doi: 10.1371/journal.pone.0151761 (PMC4798727; doi:10.1371/journal.pone.0151761)
Supplement: S1 Fig — The expression of intracellular IL-10 and surface markers CD24, CD38 and CD27 were determined by flow cytometry after in vitro stimulation of PBMC for 5h with only BFA; CpG (10μg/ml) + PIB or LPS (10μg/ml) + PIB. This cytometry plot shows (A) CD19+CD24hiCD38hi and (B) CD19+CD24hiCD27+ B10 cells after CpG + PIB stimulation. (PDF) [file pone.0151761.s001.pdf]

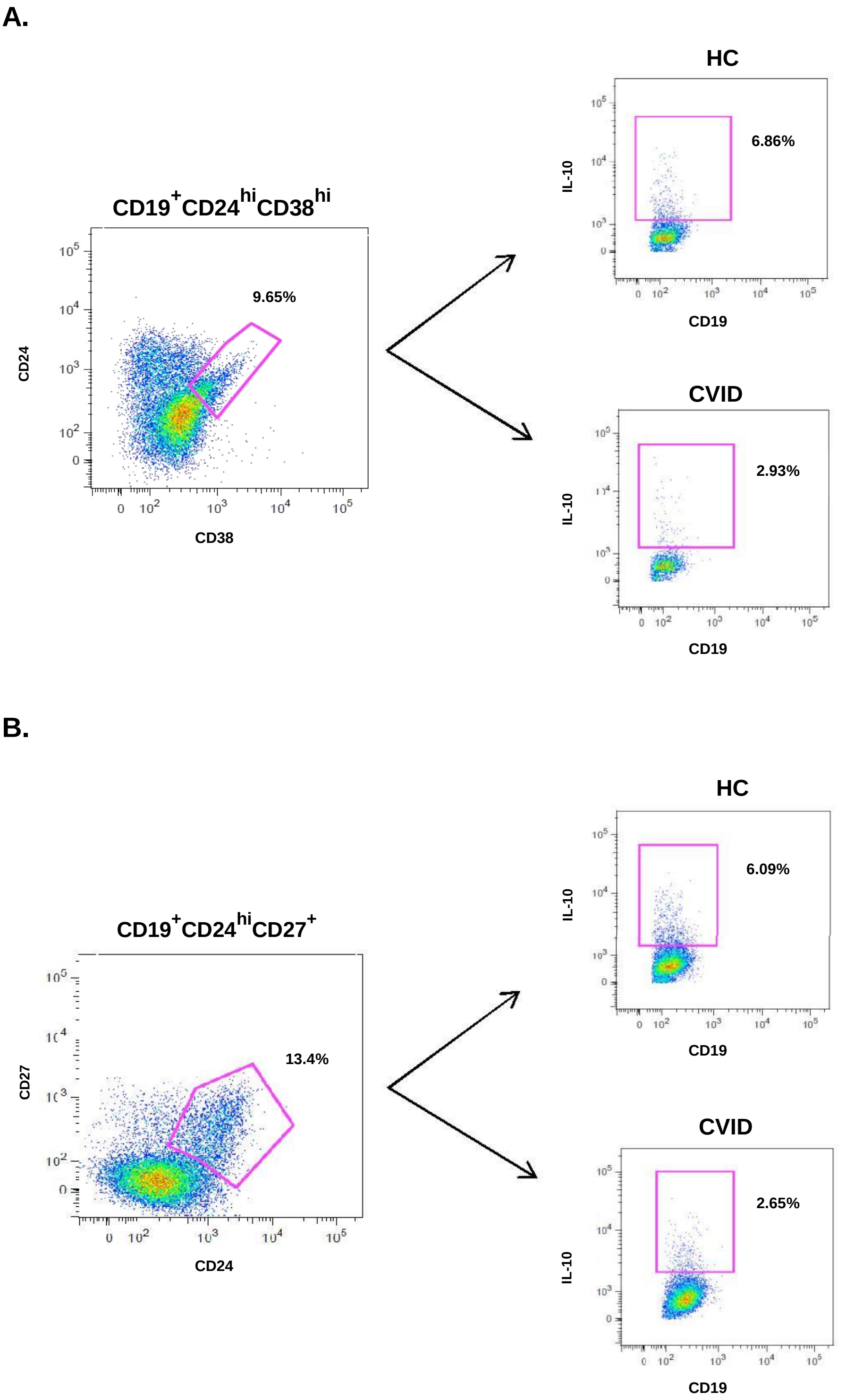

Figure S1. Representative flow cytometry plot showing the gating strategy used to identify B10 cells. The expression of intracellular IL-10 and surface markers CD24, CD38 and CD27 were determined by flow cytometry after in vitro stimulation of PBMC for 5h with only BFA; CpG (10µg/ml) + PIB or LPS (10µg/ml) + PIB. This cytometry plot shows **(A)** $CD19^{+}CD24^{hi}CD38^{hi}$ and **(B)** $CD19^{+}CD24^{hi}CD27^{+}$ B10 cells after CpG + PIB stimulation.
